# Animal and Plant Protein Food Sources in Indonesia Differ Across Socio-Demographic Groups: Socio-Cultural Research in Protein Transition in Indonesia and Malaysia

**DOI:** 10.3389/fnut.2022.762459

**Published:** 2022-02-11

**Authors:** Helda Khusun, Judhiastuty Februhartanty, Roselynne Anggraini, Elise Mognard, Yasmine Alem, Mohd Ismail Noor, Norimah Karim, Cyrille Laporte, Jean-Pierre Poulain, Pablo Monsivais, Adam Drewnowski

**Affiliations:** ^1^SEAMEO Regional Center for Food and Nutrition (RECFON)—Pusat Kajian Gizi Regional Universitas Indonesia, Jakarta, Indonesia; ^2^Taylor's Toulouse University Center, Subang Jaya, Malaysia; ^3^Faculty of Social Sciences and Leisure Management, Taylor's University, Subang Jaya, Malaysia; ^4^CERTOP UMR-CNRS 5044, University of Toulouse Jean Jaurès, Toulouse, France; ^5^Faculty of Health Sciences, Community Health Centre, Universiti Kebangsaan Malaysia, Kuala Lumpur, Malaysia; ^6^Department of Nutrition and Exercise Physiology, Elson S. Floyd College of Medicine, Washington State University, Spokane, WA, United States; ^7^Department of Epidemiology, Center for Public Health Nutrition, University of Washington, Seattle, WA, United States

**Keywords:** protein transition, animal protein, plant protein, socio-demographics, fish, Malaysia, Indonesia

## Abstract

**Background:**

Plant-based diets in lower-income countries are often associated with inadequate protein nutrition and adverse health outcomes.

**Objective:**

To examine the diversity of protein food sources, in both animal and plant, across diverse socio-demographic groups in Indonesia as compared to Malaysia.

**Design:**

The SCRiPT (Socio Cultural Research in Protein Transition) study was based on population-based samples recruited in Indonesia (*N* = 1665) and in Malaysia (*N* = 1604). Data from 24-h in-person dietary recalls in each country were used to construct the frequency counts of protein sources by food group. Protein sources were defined as fish, poultry, red meat (beef, pork, and mutton), eggs, dairy, and plants (cereals, pulses, and tubers). The percent reported frequencies for animal and plant proteins were compared across socio-demographic strata and by country. Analyses were based on one-way Anovas and general linear model regressions adjusting for covariates.

**Results:**

Animal protein frequency counts were 34% of total in Indonesia, but 50% in Malaysia's. Higher reported consumption frequencies for poultry and red meat in both countries were associated with urban living, greater modernization, and higher socioeconomic status, with stronger social gradients observed in Indonesia. Reported fish consumption was higher in Indonesia than in Malaysia. Fish was more likely to be listed by rural island populations in Indonesia and was associated with lower education and incomes. Consumption frequencies for plant-based proteins were associated with lower socio-economic status in Indonesia and in Malaysia.

**Conclusions:**

More affluent groups in both countries reported higher frequencies for meat, eggs, and dairy as opposed to fish. Greater economic development in Southeast (SE) Asia is associated with more animal protein, particularly from poultry, which may displace fish, the traditional source of high quality protein for the region.

## Introduction

Economic development in SE Asia has been accompanied by a protein transition, described as a dietary shift from plant- to animal-source proteins (1, 2). As incomes rise, plant proteins from grains, tubers, and legumes are progressively replaced by animal proteins from poultry, eggs, dairy, and red meat (3–5). Analyses of food balance sheets from the Food and Agriculture Organization (FAO) of the United Nations point to the importance of meat, eggs, and dairy in assuring the optimal protein nutrition across the low- and middle-income countries (LMIC) (6). Protein nutrition is of concern to Indonesia, which was recently downgraded to a lower middle-income country status by the World Bank (7).

Fish protein has long occupied a special place in the traditional diet of the island nations of SE Asia (8, 9). In Indonesia, food balance sheets show that the annual fish consumption has risen from an average of 10.6 kg per capita in the 1970s to 28.9 kg in 2011, with further growth projected (8). However, current estimates place per capita daily protein intake from fish in Indonesia at no more than 8.5 grams per person per day (10). Inadequate dietary diversity, low fish consumption (11), and over-reliance on starchy staples have been linked to under-nutrition, low birth weight, childhood stunting, and other nutritional problems (12). The prevalence of stunting among children <5 years in Indonesia is estimated at 30.8%, whereas the prevalence of wasting is 10.2% (7). Increasing the supply of high-quality animal protein in Indonesia has the potential to prevent stunting and iron deficiency anemia (11).

Indonesia and Malaysia are at different stages of economic development and at different phases of the protein transition (12). Total protein intakes in the neighboring Malaysia, an upper middle-income country, are in excess of recommended values (13). Per capita fish consumption in Malaysia has been estimated at 56 kg/year, closely tracked by poultry at 49 kg/year (9, 14). Based on the 2021 Global Nutrition Report (12), Malaysia is now facing the dual burden of malnutrition, with persistent childhood stunting (20.8%) now accompanied by rising obesity rates estimated at 20.9% among adult women and 15.9% among adult men.

Fish consumption among Malaysia adults was recently estimated at 168 g/day (15), with higher amounts observed among Malay ethnics and among the groups of *lower* education and incomes (1). Published analyses of SCRiPT consumption frequencies data for Malaysia (1) showed that fish consumption was associated with Malay ethnicity, rural (island) districts, older age, larger families, and lower socio-economic status. Data showing that the traditional rice and fish have come to be associated with lower socio-economic status and rural areas in Malaysia may have implications for the future of protein nutrition in Indonesia. In both countries, fish was the traditional source of high quality animal protein and associated vitamins and minerals.

The SCRiPT study, conducted in parallel and using similar instruments in Indonesia and Malaysia, was intended to characterize the diversity of protein food sources, in both animal and plant, across the socio-demographic groups (1). Socio-economic correlates of fish vs. chicken consumption were of special interest. Single-day 24-h dietary recalls from both countries were scored for the presence of protein from 10 food sources: fish, poultry (chicken) eggs, dairy, red meat (beef, pork, and mutton) and cereals, pulses, and tubers. A percentage frequency score, a proxy for the diversity of protein food sources, was derived for each study participant in Indonesia and in Malaysia samples. In each country, analyses explored protein diversity by socio-economic status, geographic location, ethnicity, and by multiple wealth and modernization indices.

## Methods

### Participants

The population-based Indonesia SCRiPT sample of adults >18 years was drawn from West Sumatra, Jakarta, West Java, East Java, Bali, and South Sulawesi. One urban and one rural district were randomly selected within each province (16). Participant selection used a multi-stage random sampling, using cluster method, proportionate-to-population size (PPS). A cluster refers to village, i.e., the lower administrative level of district, consisting of around 400–550 households. The survey team visited selected households. An eligible respondent from within the household was then selected at random. The present data were collected using in-person interviews between March and July 2018. For analysis purposes, the Indonesia SCRiPT sample was weighted by population density, by urban rural location, by age and sex. The weighting ensured that the data were for a nationally representative sample of the Indonesian population. The final analytical sample *N* = 1,665 was composed of participants from the densely populated Java Island provinces (*N* = 1,223), metropolitan Jakarta and Bali (*N* = 230), and West Sumatra and South Sulawesi (*N* = 219).

Previously described SCRiPT sampling for Malaysia (1) followed the methods developed for the 2013 Malaysian Food Barometer (MFB1) (17) and the Malaysian Adult Nutrition Surveys (MANS) conducted in 2003 and 2014 by the Ministry of Health Malaysia (18). The stratified random sampling scheme was applied to Peninsular Malaysia, Sabah, and Sarawak to provide a population-based sample of adults aged >18 years. The final analytical sample was geographically distributed as follows: Greater Kuala Lumpur (*N* = 387); Johor (*N* = 188); Sabah (*N* = 163); Sarawak (*N* = 154); Perak (*N* = 138); Kedah (*N* = 122); Penang (*N* = 81); Kelantan (*N* = 90); Pahang (*N* = 96); Terengganu (*N* = 81); Negeri Sembilan (*N* = 54); and Malacca (*N* = 50). The present data were collected using in-person interviews between March and July 2018. Questionnaire and methodology were approved by the Human Ethics Committee of Taylor's University (reference No. HEC2017/030).

### Socio-Demographic Questionnaires

Gender was coded as male and female. Age cut points were 18–35 years; 36–46 years, and >46 years. Marital status was single vs. married or partnered. Education was captured as primary or lower school, lower secondary school, higher secondary school, and college. For Indonesia, ethnicity was a mix of ethnicity and the geographical location classed into Minangkabau and other Sumatra/Malay ethnics; Betawinese; Sundanese; Javanese; Balinese; All Sulawesi ethnics; and Madurese and Others (19). Other classifications followed those used by the Central Bureau of Statistics Indonesia (20, 21). Options for ethnic origin in Malaysia were Malay, Chinese, Indian, and non-Malay Bumiputra (1, 17).

For Indonesia, a novel 13-item “wealth index,” was based on housing condition and ownership of household belongings. The 13 input variables were: house wall material, floor material, type of toilet used, sources of electricity, sources of fuel for cooking, as well as ownership of car, bicycle, motorcycle, refrigerator, mobile phone, land line phone, and television and radio. Scores were calculated based on principal component factors analysis with varimax rotation and were split into tertiles, with the lowest tertile (T1) representing least wealth. Additional questions were asked about the number of children, size of household, urbanization, and modernization. For Malaysia, monthly income per capita in Malaysian Ringgit was stratified into 4 categories as follows: 100–699; 700–1,332; 1,333–1,999; and >2,000.

### Frequency Counts Based on Dietary 24 h Recalls

Dietary intakes for Indonesia were based on 24-h recalls administered in person and analyzed using a customized version of the NutriSurvey for Windows 2007 and the latest 2017 version of the Indonesian Food Composition Table. Dietary intake data for Malaysia were based on 24-h dietary recalls administered in person and analyzed using a standard version of NutritionistPro. Single 24-h recall is an acceptable method to assess the intakes of population and groups but does not capture the habitual dietary patterns of individuals.

For both data sets, each food consumed was assigned into one or more protein categories: fish, poultry, eggs, dairy, pork, beef, mutton, cereals, pulses, and tubers. Mutton was selected because it is consumed by Hindu groups in Malaysia (1). Examples of categorization of protein sources in 24-h food recall based on the main ingredient are shown in Supplementary Table 1. The frequency count captured the presence of animal protein in the food product and/or dish, but not the amount. For example, *Nasi Goreng* (fried rice) could be assigned to multiple categories: cereals (rice), egg (egg), and poultry (chicken) or fish (shrimp or fish cake) or pulses (tofu soy). The number of category assignments was variable, depending on dish composition. To generate the frequency count metric, the number of counts in each protein source category was summed for each participant. Univariate tests of differences across socio-demographic groups were tested using one-way Anovas. Strength of the association between socio-economic variables (education, income, and urbanization) and cultural variables (ethnicity), and plant or animal protein counts were tested in general linear models adjusting for covariates. Analyses were conducted using SPSS and SAS statistical programs.

## Results

**Figure 1:**
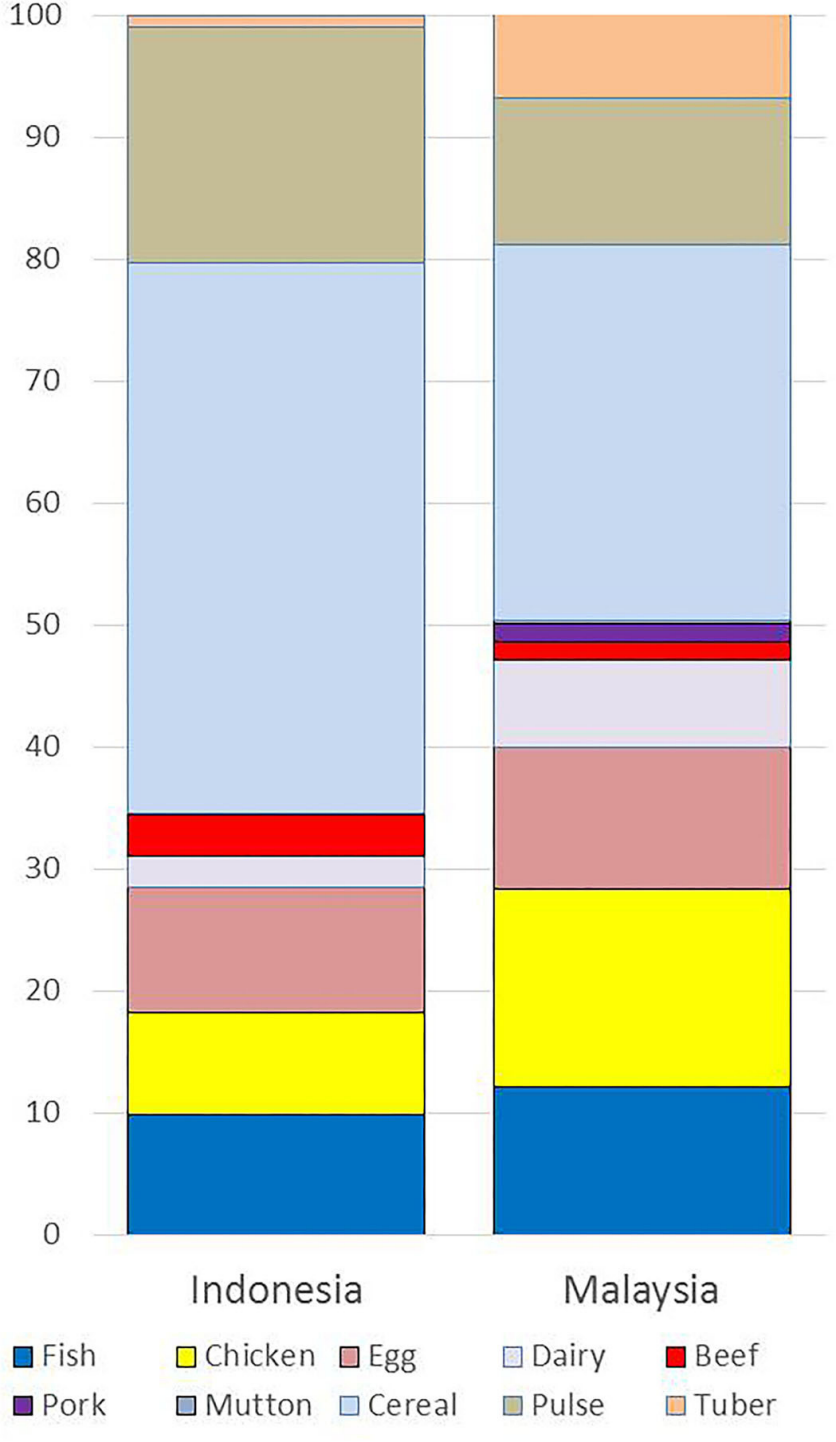
Percent frequencies of protein consumption by food source for Indonesia and Malaysia. Data for full samples: Indonesia *N* = 1,665 and Malaysia *N* = 1,604. Malaysia data based on Drewnowski et al. (1).

Figure 1 is a comparison of percent frequency counts by protein food group, based on 24-h recalls that were conducted in parallel in Indonesia and in Malaysia. For Indonesia (Figure 1), plant proteins were 65.4% of the total, mostly derived from cereals (rice) and beans. Animal proteins accounted for 35.4% of the total, and were separated further into 12% for meat and poultry, 12.8% for eggs and dairy, and 9.8% for fish. For Malaysia (Figure 1), the split between animal and plant proteins was 50:50 as previously reported (1). Plant proteins were derived from grains (rice and wheat), pulses (beans), and tubers (potatoes). The most frequent source of meat was chicken (16.2%). Red meat was relatively rare, with pork and beef accounting for only 1.5% each. Fish were 12.1% of the total and eggs and dairy 8.8% (1).

**Figure 2:**
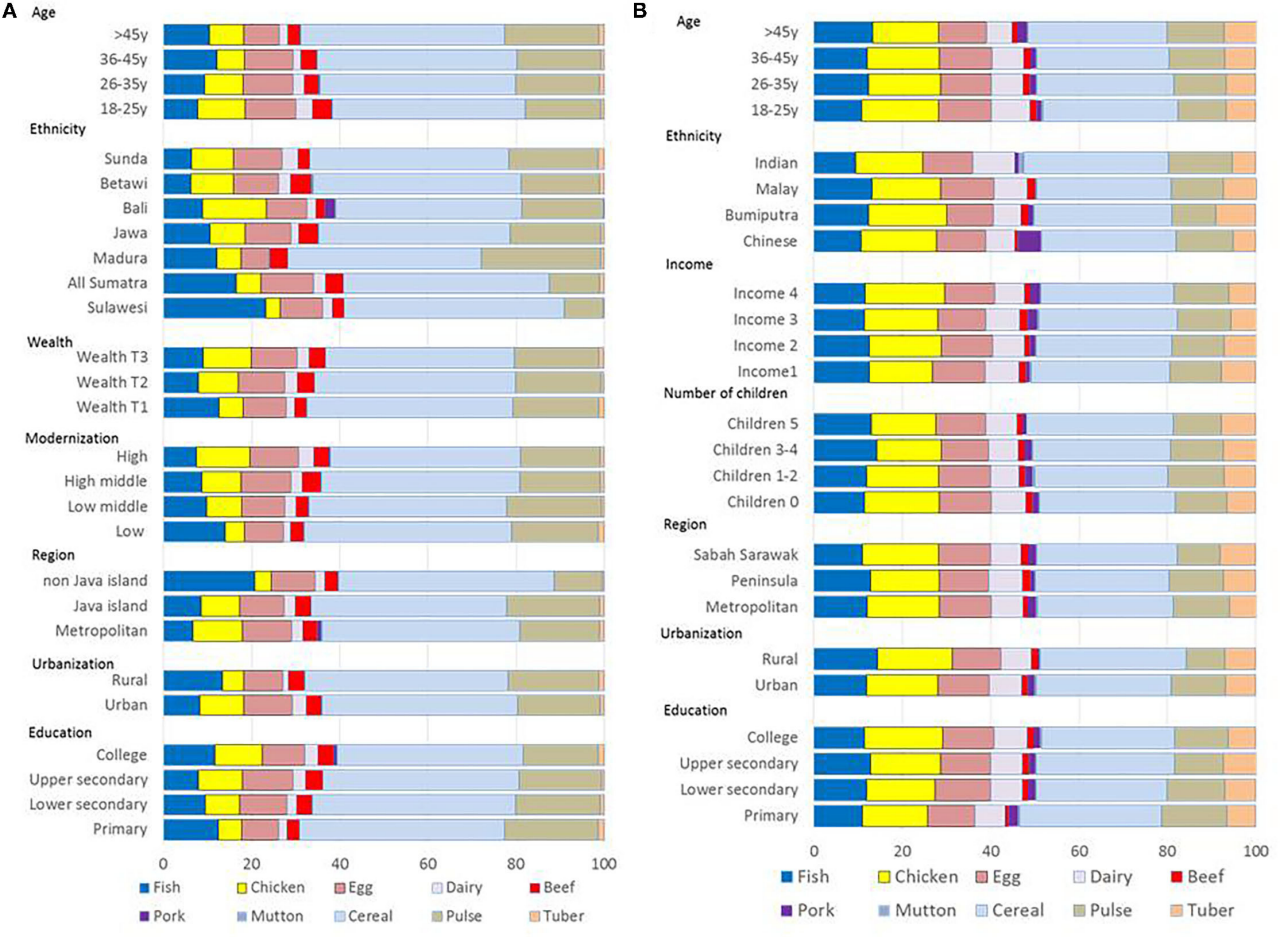
Percent frequencies of protein consumption by food source for Indonesia **(A)** and Malaysia **(B)**. Data for population subgroups by demographic variables. Malaysia data based on Drewnowski et al. (1).

Figure 2A shows the percent frequency counts by food group across socio-demographic variables for Indonesia. Bivariate analyses (Supplementary Tables 2, 3) showed significant effects of age, ethnicity, number of children, occupation, modernization, and urbanization (*p* < 0.001 for all). Higher frequencies for animal protein were associated with being single, younger, college educated, wealthier, working in professional jobs, and having fewer children (*p* < 0.001).

The socio-demographic gradient for plant protein frequencies was in the opposite direction. Higher plant protein frequencies were obtained for older adults (*p* < 0.001) working in blue collar jobs (*p* < 0.003), and for groups in the lower wealth tertile (*p* < 0.001). Higher plant protein frequencies were associated with rural settings, bigger household size, and more children (*p* < 0.001 for all).

Reported frequencies of fish consumption were a special case. There were significant effects of age, occupation, modernization, and wealth index tertiles (*p* < 0.001 for all). Higher fish frequencies were associated with older age groups, less wealth, rural settings, lower modernization, married status (*p* < 0.001), and with more children (*p* < 0.001). There was also an urban rural divide: highest fish frequency counts were obtained in South Sulawesi (22.3%) as compared to Bali (6.9%). By contrast, Bali residents reported the highest frequencies for chicken and red meat (*p* < 0.001).

Fish consumption was associated with older adults with lower income (*p* < 0.001). Younger people listed more eggs, dairy, poultry, and beef, whereas older people listed more fish. Professional occupations were associated with higher reported frequencies of poultry, beef, and pork (*p* < 0.001 for all). The effects of wealth index were significant for poultry (*p* < 0.001), dairy (*p* < 0.05), and pork (*p* < 0.01). Higher wealth was associated with lower reported consumption frequencies for fish (*p* < 0.001).

Urbanization was linked to more poultry, eggs, and dairy but less fish (*p* < 0.001 for all). Similarly, the modernization index was linked to more poultry (*p* < 0.001), egg (*p* < 0.01); dairy (*p* < 0.001), and beef (*p* < 0.05) but less fish (*p* < 0.001). By contrast, the number of children was related to more fish (*p* < 0.001) and lower frequencies for poultry (*p* < 0.05), egg (*p* < 0.01), and dairy (*p* < 0.001).

The consumption of poultry (chicken) vs. fish varied by province. Highest frequency counts for chicken (by far) were observed in Bali, while the highest counts for fish were observed in South Sulawesi and West Sumatra. Pork and mutton frequencies were restricted to Bali, whereas higher counts for beef were observed in Jakarta and East Java. Muslim religion was associated with less chicken, more beef, and no pork.

Also shown in Figure 2A are percent frequency counts for cereals (rice), legumes, and tubers. Cereals accounted for 45% of the total; legumes for 19.3%, and tubers for only 1%. Here, the socio-demographic trends were operating in the opposite direction. Older adults reported higher frequencies for both cereals and legumes (*p* < 0.05 for both). Higher education and occupation status were associated with lower consumption frequencies for cereals and legumes (*p* < 0.05 for all). Also associated with lower consumption frequencies for cereals (rice) were the wealth index (*p* < 0.001), urban setting (*p* < 0.05), and modernization (*p* < 0.001).

Data for Malaysia (1) shown in Figure 2B contrast with the Indonesia data in that the percent of animal proteins was closer to 50%. In common with Indonesia, higher reported frequencies for animal proteins were associated with higher education and incomes, though variations by ethnic group were also observed. In particular, pork consumption was associated with higher incomes and Chinese ethnicity.

Multivariate regression model (Table 1) tested the associations between ethnicity, wealth and urbanization, and the nature of protein food sources. The model adjusted for basic socio-demographics: age, gender, marital status, and number of children, and for all other variables in the Table 1. In the adjusted model, poultry and meat consumption varied with ethnicity (i.e., island location) and was associated with a higher wealth index and greater urbanization. The consumption of chicken and red meat was significantly higher in Bali than in other locations. By contrast, plant protein consumption frequencies were strongly associated with lower wealth index and rural area. Fish consumption frequencies showed a unique pattern: the main effects in the adjusted regression model were ethnicity (higher in Sumatera and Sulawesi ethnics) and rural (island) location.

**Table 1:** Multivariable linear regression analyses for percent frequencies of meat and poultry, cereals and pulses, and fish by socio-demographics.

**Variables**	***N***	**Meat and poultry**			**Cereals and pulses**			**Fish**		
**Ethnicity**		**Coeff**	**95% CI**	***p*** **-value**	**Coeff**	**95% CI**	***p*** **-value**	**Coeff**	**95% CI**	***p*** **-value**
Betawinese	73	Ref			Ref			Ref		
Balinese	143	**6.02**	**1.83; 10.21**	**0.01**	**−6.21**	**−11.27; −1.14**	**0.02**	−0.74	−4.60; 3.23	0.71
Sundanese	661	−1.04	−4.23; 2.14	0.52	−0.37	−4.22; 3.47	0.85	−1.55	−4.48; 1.39	0.30
Javanese	463	−1.19	−4.44; 2.06	0.47	−1.98	−5.91; 1.95	0.32	2.47	−0.53; 5.47	0.11
Sumatra	118	−2.01	−5.90; 1.89	0.31	**−9.13**	**−13.84; −4.43**	**0.00**	**7.20**	**3.60; 10.79**	**0.00**
Madurese	100	−1.39	−5.18; 2.40	0.47	3.65	−0.93; 8.23	0.12	2.16	−1.34; 5.66	0.23
Sulawesi	90	**−6.20**	**−10.18; −2.21**	**0.00**	**−8.87**	**−13.69; −4.05**	**0.00**	**14.22**	**10.54; 17.90**	**0.00**
**Wealth index**										
Tertile 3	553	Ref			Ref			Ref		
Tertile 2	559	−0.80	2.40; 0.81	0.33	1.61	−0.33; 3.55	0.10	−1.10	−2.58; 0.38	0.14
Tertile 1	551	**−3.41**	**5.23; −1.60**	**0.00**	**2.73**	**0.54; 4.92**	**0.02**	0.49	−1.19; 2.16	0.57
**Urbanization**										
Urban	1124	Ref			Ref			Ref		
Rural	541	**−3.44**	**−4.94; −2.00**	**0.00**	**3.06**	**1.25; 4.86**	**0.00**	**3.68**	**2.30; 5.06**	**0.00**

*Model adjusted by age, gender, marital status, number of children, and all variables in the Table. Significant values are indicated in bold*.

## Discussion

The present study used frequency counts of protein food sources from 24-h dietary recalls to assess the diversity and quality of protein nutrition in Indonesia. Frequency counts (1) are a semi-quantitative method of dietary intake assessment. The foods consumed are scored for the presence of animal and/or plant proteins but not the amount. As such, frequency counts cannot rival quantitative methods of dietary intake assessment but rather resemble the diversity scores that have been used by the FAO to assess the food security and diet quality worldwide (22). The Global Diet Quality project also uses 1 day consumption frequencies of different foods as a proxy index of overall diet quality (23).

Plant proteins, mostly from rice, were still dominant in the Indonesian diet: the relative frequencies were 65.4% for plant protein and 34.6% for animal proteins. The comparison with Malaysia is of interest, given that Malaysia is at a more advanced state of the protein transition (1, 17). As had been shown before (1), animal protein in Malaysia now accounted for 50% of the total, mostly from chicken, eggs, and dairy. In Malaysia, the most likely to list chicken, eggs, or dairy were the younger urban adults who were also least likely to list fish. Higher frequency of meat and poultry (but not fish) consumption in Malaysia was associated with higher incomes (1). Compared to chicken, the consumption of red meat (beef, pork and mutton) in Malaysia was much less common.

Fish has been the traditional source of high-quality animal protein in both Indonesia and Malaysia (8, 9). In Indonesia, rice is still at the center of most meals, accompanied by chicken, beef, vegetables, or fish (24). Based on the published reports, the preferred cooking styles in Malaysia were deep-fried fish, fish in chili gravy, fish curry, and fish cooked in coconut milk (15). The present analyses point to social and regional gradients in fish consumption that operate in both countries. In the SCRiPT Malaysian sample, the most likely to report rice and fish in 24-h dietary recall were the lower-income rural populations with more children (1). Also, in Indonesia, higher reported frequencies for rice and fish were now associated with older age, lower education and wealth, lower modernization, rural setting, and having more children. In both countries, higher poultry and meat protein consumption frequencies were reported by single, younger, and college-educated adults working in professional jobs. It would appear that the traditional SE Asian diets of rice and fish are now more frequent among the rural poor, as the higher-income urban groups report more chicken and other sources of animal protein such as meat, eggs, and dairy.

The present data are consistent with the results of a recent study on urbanization, dietary change, and traditional food practices in Indonesia (25). Based on longitudinal analyses of food expenditures, the study found higher expenditure shares for meat, eggs, and milk products, and lower expenditure shares for staple foods (25). Although traditional diets high in plant proteins continued to be dominant in both rural and urban areas, the urban Jakarta area was associated with more ‘Western’ food patterns. Expenditure shares for foods associated with the local traditional diet, such as fish and vegetables, have not decreased. While the traditional food patterns in Indonesia show a degree of resilience, further changes may be driven by the ongoing nutrition transition.

Future dietary patterns in Indonesia may be informed, if not predicted, by the experience in Malaysia. Higher incomes at the country and household levels are normally associated with a lower percentage of energy from starchy staples, as predicted by Bennett's Law (26, 27). It should also be noted that higher percentages of animal protein in the diet are usually accompanied by a higher consumption of animal fats, (28, 29). That plant proteins are replaced by animal source proteins is a component of the protein transition (1, 2). However, the selection of a particular animal protein may depend on regional resources, traditions, and culture. In Indonesia, the sources of animal protein were eggs, fish, and poultry, with less frequent beef and dairy, and virtually no pork. Chicken consumption in Indonesia is estimated at only 7.9 kg/person/year (14).

Reported meat consumption frequency was relatively low, consistent with low meat consumption reported in the Indonesia Total Diet Study (30). Unlike the Total Diet Study, the present analyses of frequency counts were able to distinguish between beef and chicken. Chicken consumption frequency was highest in Bali and lowest in South Sulawesi province (5.3%), while beef was lowest in Bali despite the higher wealth quintile. Cultural and religious factors may have been the reason. More than 80% of Balinese population were Hindu, in contrast to Java where more than 80% were Muslim. These socio-cultural differences may determine the choice of beef vs. chicken in Jakarta, Java, and Bali.

The observed social gradients can provide some insight into the future of food demand in SE Asia (26). Even though the nutrition transition has sometimes been viewed as a purely economic phenomenon (1), social and cultural factors are clearly involved as well. The observed interactions between ethnicity (or region) and wealth pointed to important differences in the adoption of “modern” diets across the provinces of Indonesia (31). In general, diets with a higher proportion of meat, poultry, and eggs, as opposed to the more traditional fish, were associated with higher socio-economic status, and younger age groups with higher education and incomes. There were also regional differences in the amounts and types of animal proteins consumed. South Sulawesi had the highest fish consumption frequency (22.3%), followed by West Sumatra (18.4%). The lowest fish consumption frequency was found in Jakarta, West Java, and Bali (around 6%). This result is consistent with data from the Indonesia Total Diet Study which showed that both Sulawesi and Sumatra provinces had a higher fish intake compared to Java and Bali (26). Sumatra and Sulawesi are both island provinces with higher percentages of rural population and lower in wealth as compared to Java and Bali.

Protein diversity by food source may provide a proxy index of protein quality and may be further linked to health outcomes (32, 33). The FAO recommends achieving amino acid balance by supplementing grain- and cereal-based diets in LMIC with small amounts of animal protein. Although protein food sources have served as proxy measures of protein quality, the present diversity score needs to be compared to other measures such as amino acid profiles and essential amino acid adequacy score.

## Conclusion

An increased consumption of animal protein foods, following an economic development, is dependent on education and income, as well as on location, ethnicity, and on social and cultural norms. In a higher income country like Malaysia, poultry, eggs, and dairy were listed more frequently than fish. Other than for island provinces, fish consumption in Indonesia may also be replaced by more poultry and dairy products. Studying the socio-cultural determinants of food selection is essential for a better understanding of food security and adequate protein nutrition in SE Asia.

## Data Availability Statement

The raw data supporting the conclusions of this article will be made available by the authors, without undue reservation.

## Ethics Statement

The studies involving human participants were reviewed and approved by Human Ethics Committee of Taylor's University (Reference No. HEC2017/030). Written informed consent was obtained from all subjects. The patients/participants provided their written informed consent to participate in this study.

## Author Contributions

AD and J-PP have conceptualized the SCRiPT study. J-PP has organized the data collection both in Malaysia and Indonesia in the framework of the Asian Food Barometer. EM, YA, MN, NK, and CL were responsible for data collection in Malaysia. HK, JF, and RA were responsible for data collection in Indonesia. EM and PM took the lead on data analysis. AD and PM took the lead on data presentation and the manuscript. All authors have contributed to the design of study protocols, reviewed, and approved the final manuscript.

## Funding

Socio-Cultural and Economic Research in Protein Transition in SE Asia: Focus on Malaysia and Indonesia (SCRiPT) survey was supported by a grant from Ajinomoto Co. Japan to Taylor's University, Kuala Lumpur, Malaysia; the International Associated Laboratory (LIA) CNRS Food Cultures and Health.

## Conflict of Interest

AD is a consultant to Ajinomoto Co. and has received grants, contacts, and honoraria from public agencies, non-profit organizations, and private entities with an interest in nutrient profiling and in assessing the nutrient density of food patterns and the total diet. The remaining authors declare that the research was conducted in the absence of any commercial or financial relationships that could be construed as a potential conflict of interest.

## Publisher's Note

All claims expressed in this article are solely those of the authors and do not necessarily represent those of their affiliated organizations, or those of the publisher, the editors and the reviewers. Any product that may be evaluated in this article, or claim that may be made by its manufacturer, is not guaranteed or endorsed by the publisher.
