# Supplementary material for: Animal and Plant Protein Food Sources in Indonesia Differ Across Socio-Demographic Groups: Socio-Cultural Research in Protein Transition in Indonesia and Malaysia
Source: Front Nutr. 2022 Feb 11;9:762459. doi: 10.3389/fnut.2022.762459 (PMC8886573; doi:10.3389/fnut.2022.762459)
Supplement: Supplementary file 1 [file Table_1.DOCX]

| Supplemental Table 1. Indonesia - Categorization of protein sources in 24h food recall based on the main ingredient. | | |
| --- | --- | --- |
| **No.** | **Menu** | **Protein sources** |
|  | White rice | Cereals |
|  | Fried rice with egg and chicken | Cereals, eggs, poultry, pulses |
|  | Chicken porridge | Cereals, poultry, pulses |
|  | Instant noodle with egg | Cereals, eggs |
|  | Gado-gado (mixed vegetables with egg and peanut sauce) | Eggs, pulses |
|  | Fried chicken/duck | Poultry |
|  | Chicken nugget | Poultry |
|  | Opor/kare ayam/soto ayam (chicken with coconut milk) | Poultry |
|  | Chicken satai | Poultry, pulses |
|  | Fried egg | Eggs |
|  | Fried catfish, tilapia fish, tuna | Fish |
|  | Anchovy | Fish |
|  | Tongseng (beef/mutton with coconut milk) | Beef/mutton, pulses |
|  | Coto makasar (beef/mutton with coconut milk) | Beef/mutton, pulses |
|  | Rawon (beef soup) | Beef |
|  | Rendang | Beef |
|  | Meatball | Beef |
|  | Tempeh, Tofu | Pulses |
|  | Milk powder, Ready to drink milk, Ice cream | Dairy |
|  | Martabak (stuffed eggs) | Cereals, eggs, poultry/beef |
|  | Siomay bandung/batagor/seblak (fried dumpling with peanut sauce) | Cereals, fish, pulses |
|  | Pempek (fish cake) | Cereal, fish |
|  | Lemper (glutinous rice filled with shredded chicken) | Cereal, poultry |
|  | Kappurung (sago dish served with fish) | Cereals, fish |
|  | Barobbo (porridge cooked with egg and vegetable) | Eggs, cereals |
|  | Danke (fermented milk) | Dairy |
