# Supplementary material for: Animal and Plant Protein Food Sources in Indonesia Differ Across Socio-Demographic Groups: Socio-Cultural Research in Protein Transition in Indonesia and Malaysia
Source: Front Nutr. 2022 Feb 11;9:762459. doi: 10.3389/fnut.2022.762459 (PMC8886573; doi:10.3389/fnut.2022.762459)
Supplement: Supplementary file 2 [file Table_2.DOCX]

| **Supplemental Table 2. Percent reported consumption frequencies of animal protein sources (mean and SEM) from 24h intakes by socio-demographic variables and protein type. Animal proteins are coded as fish, poultry, egg, dairy, beef, pork, mutton. P-values are for univariate analyses based on one-way ANOVA. Data are for Indonesia SCRiPT study** | | | | | | | | | |
| --- | --- | --- | --- | --- | --- | --- | --- | --- | --- |
| **Socio-demographic Characteristics** | **Indonesia** | | **Fish** | **Poultry** | **Egg** | **Dairy** | **Beef** | **Pork** | **Mutton** |
|  | **Count** | **%** | **Mean ± SEM** | **Mean ± SEM** | **Mean ± SEM** | **Mean ± SEM** | **Mean ± SEM** | **Mean ± SEM** | **Mean ± SEM** |
| **All** | 1665 | 100 | 9.81 ± 0.32 | 8.43 ± 0.28 | 10.32 ± 0.28 | 2.46 ± 0.15 | 3.44 ± 0.18 | 0.13 ± 0.05 | 0.04 ± 0.02 |
| **Age groups (years)** |  |  |  |  |  |  |  |  |  |
| 18-25 | 354 | 21.2 | 7.66 ± 0.61 | 10.76 ± 0.67 | 11.58 ± 0.67 | 3.80 ± 0.45 | 4.35 ± 0.40 | 0.10 ± 0.09 | 0.03 ± 0.04 |
| 26-35 | 476 | 28.6 | 9.31 ± 0.57 | 8.76 ± 0.53 | 11.33 ± 0.51 | 2.53 ± 0.28 | 3.26 ± 0.29 | 0.21 ± 0.12 | 0.06 ± 0.04 |
| 36-45 | 337 | 20.3 | 12.05 ± 0.77 | 6.32 ± 0.52 | 10.97 ± 0.60 | 1.76 ± 0.25 | 3.60 ± 0.44 | 0.15 ± 0.09 | 0.06 ± 0.04 |
| 46 and above | 498 | 29.9 | 10.31 ± 0.60 | 7.88 ± 0.53 | 8.03 ± 0.47 | 1.93 ± 0.23 | 2.86 ± 0.35 | 0.06 ± 0.05 | 0.02 ± 0.02 |
| *P-Value* |  |  | **<0.001***** | **<0.001***** | **<0.001***** | **<0.001***** | **0.034**** | 0.645 | 0.699 |
| **Ethnicity** |  |  |  |  |  |  |  |  |  |
| All Sumatera | 118 |  | 16.49 ± 1.20 | 5.59 ± 0.89 | 12.01 ± 1.13 | 2.65 ± 0.55 | 4.02 ± 0.76 | 0.00 ± 0.00 | 0.08 ± 0.10 |
| Betawi | 73 |  | 6.13 ± 1.14 | 9.64 ± 1.26 | 10.28 ± 1.16 | 2.78 ± 0.73 | 4.62 ± 0.92 | 0.17 ± 0.20 | 0.26 ± 0.19 |
| Sunda | 661 |  | 6.26 ± 0.37 | 9.61 ± 0.45 | 10.97 ± 0.41 | 3.61 ± 0.29 | 2.70 ± 0.22 | 0.00 ± 0.00 | 0.00 ± 0.00 |
| Jawa | 463 |  | 10.59 ± 0.64 | 7.85 ± 0.52 | 10.54 ± 0.58 | 1.59 ± 0.24 | 4.45 ± 0.39 | 0.00 ± 0.00 | 0.05 ± 0.03 |
| Bali | 90 |  | 8.76 ± 1.38 | 14.60 ± 1.48 | 9.09 ± 1.14 | 2.08 ± 0.52 | 2.02 ± 0.60 | 2.28 ± 0.81 | 0.21 ± 0.20 |
| All Sulawesi Ethnics | 100 |  | 23.15 ± 1.67 | 3.34 ± 0.79 | 9.63 ± 1.06 | 2.23 ± 0.59 | 2.64 ± 0.65 | 0.00 ± 0.00 | 0.00 ± 0.00 |
| Madura | 143 |  | 12.06 ± 1.22 | 5.50 ± 0.96 | 6.31 ± 0.92 | 0.28 ± 0.15 | 4.06 ± 0.95 | 0.00 ± 0.00 | 0.00 ± 0.00 |
| Others | 18 |  | 4.84 ± 2.33 | 14.33 ± 3.91 | 11.76 ± 2.72 | 1.18 ± 0.99 | 2.82 ± 1.38 | 0.00 ± 0.00 | 0.00 ± 0.00 |
| *P-Value* |  |  | **<0.001***** | **<0.001***** | **0.001***** | **<0.001***** | **0.002***** | **<0.001***** | 0.27 |
| **Wealth index** |  |  |  |  |  |  |  |  |  |
| T1 (Low Wealth) | 551 | 33.2 | 12.56 ± 0.62 | 5.38 ± 0.47 | 9.87 ± 0.47 | 1.86 ± 0.24 | 2.76 ± 0.31 | 0.04 ± 0.04 | 0.02 ± 0.02 |
| T2 (Medium) | 559 | 33.6 | 7.95 ± 0.50 | 9.00 ± 0.47 | 10.57 ± 0.47 | 2.83 ± 0.25 | 3.86 ± 0.32 | 0.09 ± 0.08 | 0.04 ± 0.03 |
| T3 (High Wealth) | 553 | 33.2 | 8.93 ± 0.52 | 10.91± 0.51 | 10.42 ± 0.50 | 2.70 ± 0.30 | 3.71 ± 0.32 | 0.27 ± 0.11 | 0.06 ± 0.04 |
| *P-Value* |  |  | **<0.001***** | **<0.001***** | 0.487 | **0.019**** | **0.03**** | 0.100 | 0.604 |
| **Modernization** |  |  |  |  |  |  |  |  |  |
| Low | 380 | 22.8 | 13.89 ± 0.78 | 4.46 ± 0.55 | 8.93 ± 0.55 | 1.43 ± 0.25 | 3.01 ± 0.41 | 0.10 ± 0.11 | 0.00 ± 0.00 |
| Low middle | 433 | 26 | 9.68 ± 0.62 | 7.97 ± 0.55 | 9.81 ± 0.55 | 2.52 ± 0.30 | 2.91 ± 0.35 | 0.04 ± 0.05 | 0.06 ± 0.04 |
| High middle | 462 | 27.7 | 8.59 ± 0.55 | 8.99 ± 0.50 | 11.29 ± 0.50 | 2.54 ± 0.25 | 4.29 ± 0.36 | 0.11 ± 0.09 | 0.02 ± 0.02 |
| High | 390 | 23.4 | 7.44 ± 0.55 | 12.13± 0.64 | 11.12 ± 0.63 | 3.33 ± 0.41 | 3.45 ± 0.34 | 0.28 ± 0.12 | 0.09 ± 0.05 |
| *P-Value* |  |  | **<0.001***** | **<0.001***** | **0.008***** | **<0.001***** | **0.024**** | 0.324 | 0.233 |
| **Region** |  |  |  |  |  |  |  |  |  |
| Metropolitan Jakarta | 230 | 13.8 | 6.71 ± 0.70 | 11.17 ± 0.83 | 11.20 ± 0.71 | 2.48 ± 0.37 | 3.29 ± 0.47 | 0.87 ± 0.32 | 0.19 ± 0.10 |
| Java Island Provinces | 1216 | 73 | 8.45 ± 0.34 | 8.73 ± 0.34 | 10.24 ± 0.33 | 2.49 ± 0.18 | 3.57 ± 0.22 | 0.00 ± 0.00 | 0.01 ± 0.01 |
| Non-Java Island | 219 | 13.2 | 20.61 ± 1.04 | 3.84 ± 0.56 | 9.87 ± 0.79 | 2.31 ± 0.40 | 2.89 ± 0.47 | 0.07 ± 0.10 | 0.04 ± 0.05 |
| *P-Value* |  |  | **<0.001***** | **<0.001***** | 0.405 | 0.922 | 0.435 | **<0.001***** | **0.002***** |
| **Region Provinces** |  |  |  |  |  |  |  |  |  |
| West Sumatera | 92 | 5.5 | 18.35 ± 1.33 | 5.09 ± 0.94 | 11.19 ± 1.33 | 2.30 ± 0.60 | 3.79 ± 0.84 | 0.00 ± 0.00 | 0.01 ± 0.12 |
| Jakarta | 158 | 9.5 | 6.64 ± 0.81 | 8.87 ± 0.87 | 11.74 ± 0.85 | 2.60 ± 0.47 | 4.28 ± 0.64 | 0.08 ± 0.09 | 0.16 ± 0.10 |
| West Java | 683 | 41 | 6.72 ± 0.38 | 9.21 ± 0.44 | 11.25 ± 0.45 | 3.43 ± 0.28 | 2.48 ± 0.20 | 0.00 ± 0.00 | 0.00 ± 0.00 |
| East Java | 533 | 32 | 10.68 ± 0.60 | 8.13 ± 0.52 | 8.93 ± 0.47 | 1.28 ± 0.21 | 4.97 ± 0.42 | 0.00 ± 0.00 | 0.03 ± 0.02 |
| Bali | 72 | 4.3 | 6.89 ± 1.36 | 16.23 ± 1.67 | 10.03 ± 1.29 | 2.22 ± 0.58 | 1.10 ± 0.50 | 2.62 ± 0.96 | 0.26 ± 0.25 |
| South Sulawesi | 127 | 7.6 | 22.29 ± 1.51 | 2.94 ± 0.67 | 8.92 ± 0.96 | 2.31 ± 0.54 | 2.24 ± 0.54 | 0.12 ± 0.16 | 0.00 ± 0.00 |
| *P-Value* |  |  | **<0.001***** | **<0.001***** | **0.03**** | **<0.001***** | **<0.001***** | **<0.001***** | **0.010**** |
| **Urbanization** |  |  |  |  |  |  |  |  |  |
| Urban | 1124 | 67.5 | 8.13 ± 0.35 | 10.11 ± 0.36 | 11.05 ± 0.35 | 3.02 ± 0.20 | 3.43 ± 0.21 | 0.10 ± 0.04 | 0.04 ± 0.02 |
| Rural | 541 | 32.5 | 13.30 ± 0.62 | 4.94 ± 0.43 | 8.81 ± 0.46 | 1.30 ± 0.19 | 3.47 ± 0.36 | 0.19 ± 0.11 | 0.04 ± 0.03 |
| *P-Value* |  |  | **<0.001***** | **<0.001***** | **<0.001***** | **<0.001***** | 0.914 | 0.389 | 0.983 |
| **Education** |  |  |  |  |  |  |  |  |  |
| Primary or lower | 447 | 26.9 | 12.40 ± 0.68 | 5.35 ± 0.52 | 8.42 ± 0.53 | 1.78 ± 0.23 | 2.81 ± 0.38 | 0.01 ± 0.04 | 0.02 ± 0.02 |
| Lower secondary school | 302 | 18.1 | 9.47 ± 0.76 | 7.73 ± 0.64 | 10.76 ± 0.62 | 2.24 ± 0.33 | 3.41 ± 0.42 | 0.08 ± 0.08 | 0.11 ± 0.06 |
| Upper secondary school | 727 | 43.7 | 7.90 ± 0.43 | 10.00 ± 0.41 | 11.50 ± 0.44 | 2.84 ± 0.26 | 3.79 ± 0.27 | 0.11 ± 0.06 | 0.02 ± 0.02 |
| College / University | 188 | 11.3 | 11.62 ± 0.99 | 10.77 ± 0.99 | 9.59 ± 0.67 | 2.99 ± 0.46 | 3.69 ± 0.54 | 0.57 ± 0.28 | 0.09 ± 0.09 |
| *P-Value* |  |  | **<0.001***** | **<0.001***** | **<0.001***** | **0.021**** | 0.170 | **0.006***** | 0.179 |
|  |  |  |  |  |  |  |  |  |  |
| ^1^ One-Way ANOVA; * 0.05<P<0.1; ** 0.01<P<0.05; ***0.000<P<0.01; T1-3: Tertile of wealth Index, 1~lowest and 3 ~ highest | | | | | | | | | |
