# Supplementary material for: Animal and Plant Protein Food Sources in Indonesia Differ Across Socio-Demographic Groups: Socio-Cultural Research in Protein Transition in Indonesia and Malaysia
Source: Front Nutr. 2022 Feb 11;9:762459. doi: 10.3389/fnut.2022.762459 (PMC8886573; doi:10.3389/fnut.2022.762459)
Supplement: Supplementary file 3 [file Table_3.DOCX]

| **Supplemental Table 3. Percent reported frequencies of plant protein sources (Mean and SEM) from 24h intakes by socio-demographics and protein type. Plant proteins are divided into cereals, legumes and tubers. Data are for Indonesia SCRiPT study.** | | | | | |
| --- | --- | --- | --- | --- | --- |
| **Socio-demographic Characteristics** | **Indonesia** | | **Cereals** | **Legumes** | **Tubers** |
|  | **Count** | **%** | **Mean ± SEM** | **Mean ± SEM** | **Mean ± SEM** |
| **All** | 1665 | 100 | 45.04 ± 0.32 | 19.31 ± 0.40 | 1.01 ± 0.10 |
| **Age groups (years)** |  |  |  |  |  |
| 18-25 | 354 | 21.2 | 43.76 ± 0.69 | 17.23 ± 0.82 | 0.71 ± 0.15 |
| 26-35 | 476 | 28.6 | 44.40 ± 0.65 | 19.02 ± 0.75 | 1.12 ± 0.20 |
| 36-45 | 337 | 20.3 | 45.30 ± 0.62 | 19.03 ± 0.85 | 0.76 ± 0.17 |
| >45 | 498 | 29.9 | 46.38 ± 0.61 | 21.26 ± 0.79 | 1.27 ± 0.24 |
| *P-Value* |  |  | **0.022**** | **0.005***** | 0.163 |
| **Ethnicity** |  |  |  |  |  |
| All Sumatera | 118 |  | 46.73 ± 1.05 | 11.40 ± 1.21 | 1.03 ± 0.32 |
| Betawi | 73 |  | 47.28 ± 1.42 | 17.69 ± 1.67 | 1.15 ± 0.41 |
| Sunda | 661 |  | 45.15 ± 0.52 | 20.36 ± 0.57 | 1.34 ± 0.20 |
| Jawa | 463 |  | 43.60 ± 0.59 | 20.46 ± 0.80 | 0.86 ± 0.18 |
| Bali | 90 |  | 42.28 ± 1.40 | 18.46 ± 1.82 | 0.22 ± 0.19 |
| All Sulawesi Ethnics | 100 |  | 49.97 ± 1.39 | 8.74 ± 1.18 | 0.29 ± 0.20 |
| Madura | 143 |  | 43.93 ± 1.15 | 27.12 ± 1.84 | 0.74 ± 0.28 |
| Others | 18 |  | 52.91 ± 3.03 | 10.45 ± 2.90 | 1.70 ± 1.21 |
| *P-Value* |  |  | **<0.001***** | **<0.001***** | 0.107 |
| **Wealth index** |  |  |  |  |  |
| T1 (Low Wealth) | 551 | 33.2 | 46.70 ± 0.55 | 19.50 ± 0.76 | 1.30 ± 0.23 |
| T2 (Medium) | 559 | 33.6 | 45.59 ± 0.54 | 19.26 ± 0.66 | 0.70 ± 0.12 |
| T3 (High Wealth) | 553 | 33.2 | 42.82 ± 0.58 | 19.17 ± 0.68 | 1.02 ± 0.17 |
| *P-Value* |  |  | **<0.001***** | 0.940 | **0.059*** |
| **Modernization** |  |  |  |  |  |
| Low | 380 | 22.8 | 47.21 ± 0.70 | 19.60 ± 0.93 | 1.38 ± 0.29 |
| Low middle | 433 | 26 | 44.94 ± 0.60 | 21.41 ± 0.84 | 0.68 ± 0.18 |
| High middle | 462 | 27.7 | 44.93 ± 0.60 | 18.23 ± 0.70 | 1.02 ± 0.17 |
| High | 390 | 23.4 | 43.17 ± 0.70 | 17.98 ± 0.77 | 1.01 ± 0.19 |
| *P-Value* |  |  | **<0.001***** | **0.009***** | 0.120 |
|  |  |  |  |  |  |
| **Region** |  |  |  |  |  |
| Metropolitan | 230 | 13.8 | 44.88 ± 0.86 | 18.17 ± 1.01 | 1.02 ± 0.27 |
| Java Island Provinces | 1216 | 73 | 44.37 ± 0.38 | 21.02 ± 0.48 | 1.10 ± 0.13 |
| Non-Java Island Provinces | 219 | 13.2 | 48.98 ± 0.87 | 10.99 ± 0.89 | 0.42 ± 0.15 |
| *P-Value* |  |  | **<0.001***** | **<0.001***** | **0.085*** |
| **Urbanization** |  |  |  |  |  |
| Urban | 1124 | 67.5 | 44.51 ± 0.39 | 18.69 ± 0.46 | 0.90 ± 0.11 |
| Rural | 541 | 32.5 | 46.14 ± 0.59 | 20.59 ± 0.80 | 1.23 ± 0.22 |
| *P-Value* |  |  | **0.018**** | **0.028**** | 0.144 |

^1^ One-Way ANOVA; * 0.05<P<0.1; ** 0.01<P<0.05; ***0.000<P<0.01; T1-3: Tertile of wealth Index, 1~lowest and 3 ~ highest
